# Improving outcomes of AF ablation by integrated personalized lifestyle interventions: rationale and design of the prevention to improve outcomes of PVI (POP) trial

**DOI:** 10.1007/s00392-023-02185-5

**Published:** 2023-03-31

**Authors:** Jasper Vermeer, Tineke Vinck, Bianca de Louw, Stacey Slingerland, Marcel van ’t Veer, Marta Regis, Jos-marien Jansen, Edwin van den Heuvel, Lukas Dekker

**Affiliations:** 1 Department of Cardiology, Catharina Hospital Eindhoven, Michelangelolaan 2, 5623 EJ Eindhoven, The Netherlands; 2 Department of Electrical Engineering, Eindhoven University of Technology, Eindhoven, The Netherlands; 3 Department of Biomedical Engineering, Eindhoven University of Technology, Eindhoven, The Netherlands; 4 Department of Mathematics and Computer Science, Eindhoven University of Technology, Eindhoven, The Netherlands; 5 Philips Experience Design, Eindhoven, The Netherlands

**Keywords:** Atrial fibrillation, Lifestyle intervention, Nurse-led care, Pulmonary vein isolation, Randomized controlled trial, Risk factor management

## Abstract

Progression of atrial fibrillation (AF) and outcomes of ablation therapy are strongly affected by modifiable risk factors. Although previous studies show beneficial effects of modifying single risk factors, there is lack of evidence from randomized controlled trials on the effects of integrated AF lifestyle programmes. The POP trial is designed to evaluate the clinical outcomes of a dedicated nurse-led AF lifestyle outpatient clinic in patients with symptomatic AF. This study is a prospective, 1:1 randomized, single centre, investigator-initiated clinical trial in 150 patients with paroxysmal or persistent AF referred for a first pulmonary vein isolation (PVI). Prior to the ablation, patients in the intervention group receive a personalized risk factor treatment programme in a specialized, protocolized, nurse-led outpatient clinic. Patient education and durable lifestyle management is promoted with an e-health platform. Patients in the control group receive standard care by cardiologists before ablation. The primary endpoint is the number of hospitalizations for re-ablation and cardioversion, with a follow-up of 12 months after ablation. Secondary endpoints include mortality, number of acute ischemic events, stroke or hospitalizations for heart failure, quality of life, number of ablations cancelled because of symptom reduction, and ablation success rate at 12 months. Determinants of patient and staff experience are explored and a cost-effectiveness analysis is included. The POP trial will help ascertain the efficacy and cost-effectiveness of an integrated technology-supported lifestyle therapy in patients with symptomatic AF. The trial is funded by the Netherlands Organisation for Health Research and Development [10070012010001]. Home sleep apnoea testing devices were provided by Itamar Medical, Ltd.

*ClinicalTrials.gov Identifier* NCT05148338.

**Graphical abstract:**

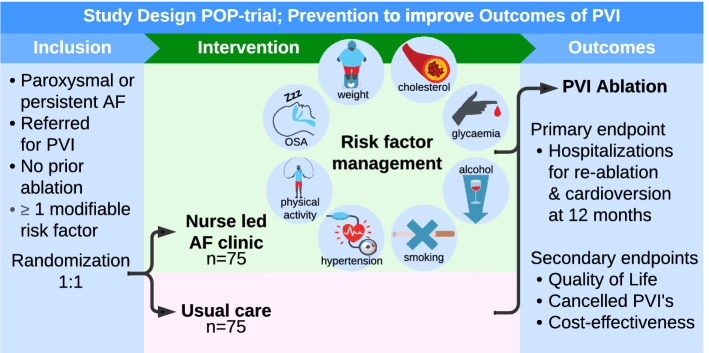

*AF* atrial fibrillation, *OSA* obstructive sleep apnoea, *PFA* pulsed field ablation, *PVI* pulmonary vein isolation.

## Background and rationale

The management of atrial fibrillation (AF) is complex and many patients experience recurrence of symptoms after an invasive treatment with catheter ablation [1]. Although general outcomes of AF ablation have improved over the years, due to innovations of ablation techniques, recurrence of arrhythmias is still experienced by at least one in five patients [2, 3]. The prevalence of AF in Europe is currently estimated at 8 million and is expected to rise to 10 million in a decade [4]. Therefore, more effective therapeutic strategies are required.

Several lifestyle risk factors have previously been identified as promoters of AF. These risk factors include obesity, physical inactivity, sleep apnoea, hypertension, smoking, alcohol use and diabetes mellitus [5–9]. In patients with body mass index (BMI) above 27 kg/m^2^, weight loss and general lifestyle advice leads to cardiac reverse remodelling and a reduction in AF symptom burden [10]. Furthermore, sustained weight loss reverses the natural progression of AF and improves arrhythmia-free survival [11, 12]. Likewise, physical inactivity independently increases the risk of AF [13]. Individuals with better cardiorespiratory fitness have a decreased risk of AF recurrence and experience less severe symptoms [14, 15]. In addition, obstructive sleep apnoea (OSA) has a severe, negative impact on AF treatment. Patients receiving OSA treatment are less likely to progress to more permanent forms of AF [16]. OSA treatment with continuous positive airway pressure (CPAP) significantly decreases AF recurrence rates after pulmonary vein isolation (PVI) [17, 18]. Comparably, beneficial AF outcomes are observed if hypertension is strictly managed, if tobacco use is quitted or if alcohol consumption is decreased [19–21].

The observational ARREST-AF and LEGACY trials clearly demonstrate beneficial effects on ablation outcomes when the management of different risk factors is combined [11, 22]. However, these trials are limited, as they only included patients with BMI above 27 kg/m^2^ and the main focus of intervention was losing weight. Besides, more than half of the included patients had sleep indices matching severe OSA. This OSA rate is higher than in comparable AF cohorts and might explain the beneficial effects of losing weight. Furthermore, participants chose their own treatment allocation, possibly leading to bias to a more motivated and healthy intervention group. It is unclear whether outcomes are generalizable to all patients with lifestyle risk factors referred for AF ablation.

The AHA 2020 scientific statement and ESC/EHRA 2020 guidelines for atrial fibrillation include recommendations on lifestyle focussed treatment as part of the management of AF [4, 23]. However, all the evidence stems from studies treating a single risk factor or from observational studies. In patients referred for AF ablation, the uncertainty in OSA prevalence has led to restrained recommendations. OSA screening is not part of the standard work-up and may only be considered if the cardiologist has a high suspicion for OSA. Evidence is lacking from randomized controlled trials on the effectiveness of an integrated lifestyle and OSA screening programme prior to ablation.

Integrated care reduces morbidity and mortality of AF patients if care is provided by well-trained nurses, because adherence to guidelines is stricter and patient education is more personalized [24–27]. However, this reduction is not as evident in centres that are less experienced with nurse-led care [28]. Before the start of this study, the POP researchers explored patients’ expectations regarding the outpatient clinic on topics, such as behaviour change and education, which led to the creation of different care programmes for the respective risk factors. Specialized nurses were then trained on how to provide direct counselling for AF patients and on how to use the different care programmes.

The goal of the POP trial is to ascertain whether in subjects with a high burden of AF and at least one treatable lifestyle risk factor, implementation of an integral AF clinic, focusing on lifestyle improvement, OSA screening, and on patient education, is beneficial for ablation outcomes. This trial aims to provide more certainty to the current limited evidence regarding the implementation of personalized integrated treatment in current AF care.

## Methods

### Trial design

**Fig. 1:**
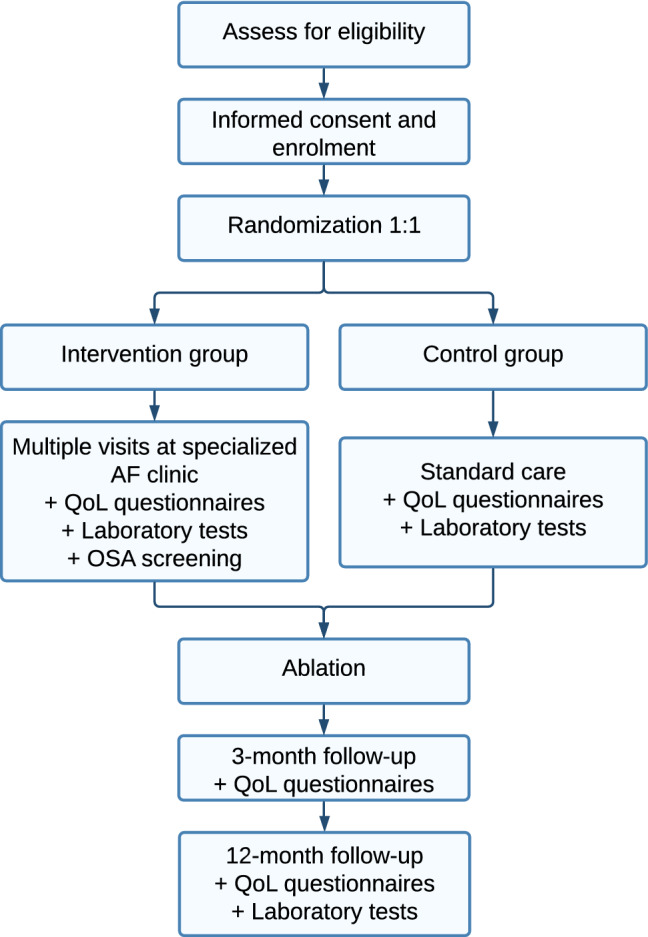
Study flowchart of the POP trial (prevention to improve outcomes after PVI). *AF* atrial fibrillation, *QoL* quality of life, *OSA* obstructive sleep apnoea

The POP study is a prospective, open-label, single centre, investigator-initiated, randomized controlled clinical trial (NCT05148338). Eligible patients referred for catheter ablation by other cardiologists in non-interventional hospitals will be randomly assigned in a 1:1 ratio to usual current temporary care or an elaborate lifestyle intervention. If a patient experiences a major reduction in AF symptoms during the pre-procedural work-up, the cardiologist will counsel the patient whether the ablation should be cancelled. Patients from both study groups will undergo a PVI according to common clinical practise. After the ablation or from the moment of the decision of the cancellation of ablation, patients will be followed for 12 months. Follow-up visits will be performed at 3 and 12 months after ablation and consist of a physical examination next to laboratory tests and quality of life questionnaires. A detailed study flow chart is shown in Fig. 1. The trial is non-blinded, since blinding is not possible, given that the treating physician is involved in the multidisciplinary team.

### Study population

The study will enrol patients with paroxysmal or persistent AF referred for their first catheter ablation. Detailed inclusion and exclusion criteria are displayed in Table 1.

**Table 1:** Inclusion and exclusion criteria

Inclusion criteria	Exclusion criteria
Age: 18–75 years Paroxysmal or persistent (shorter than 1 year) AF Referral for first catheter ablation At least one of the following risk factors: BMI ≥ 27 kg/m^2^ LDL-cholesterol > 2.6 mmol/L or total cholesterol > 5.0 mmol/L Hypertension (blood pressure > 130/90 mmHg) Diabetes mellitus with HbA1c ≥ 53 mmol/mol Active smoking Alcohol use > 14 equivalent units of alcohol/week Informed consent	Long-standing persistent AF (persistent for more than 1 year) Permanent AF Asymptomatic AF Prior catheter ablation Paroxysmal AF consisting of one episode with a reversible cause (e.g., fever, surgery, thyroid crisis, ischemic) Severe valvular heart disease Prior or soon foreseen implantation of cardiac device, such as pacemaker or internal cardioverter defibrillator Acute coronary syndrome < 3 months before inclusion Unstable heart failure NYHA class IV, or heart failure necessitating admission < 3 months before inclusion Cardiac surgery < 3 months before inclusion or planned cardiac surgery Malignancy Life expectancy < 1 year Pregnancy

*AF* atrial fibrillation, *BMI* body mass index, *LDL* low-density lipoprotein, *HbA1c* glycohemoglobin, *NYHA* New York Heart Association

The study will be conducted in accordance with the declaration of Helsinki, the good clinical practice, and the applicable regulatory requirements [29]. The locally appointed medical ethics committee has approved the research protocol. All participants will be informed by the investigator and are required to give their written informed consent prior to enrolment.

### Lifestyle interventions

Patients in the lifestyle intervention group will undergo an elaborate risk factor assessment at the nurse-led outpatient clinic. This assessment involves measuring body weight and length, blood pressure, a blood test for cholesterol and diabetes, and a home sleep apnoea test (HSAT). The HSAT is performed with a WatchPAT™ 300, which has previously been validated in a cohort of AF patients [30]. Furthermore, lifestyle behaviour such as physical inactivity, alcohol consumption and smoking are assessed in a structural interview. The risk factor definitions and treatment targets are presented in Table 2. If there are too many risk factors to be treated in a reasonable way for the patient (e.g., it would take too much time and effort), the physician will decide which risk factors will be treated, together with the patient in a shared-decision making approach. During the lifestyle intervention, patients will have approximately 3–5 physical appointments with the nurse–practitioner as well as a 10-min consultation with the cardiologist. Further additional consultations for risk interventions will take place as described in Table 2. The ablation will be scheduled when treatment targets are reached, or, if targets are not reached, at maximally 6 months after study enrolment.

**Table 2:** Risk factor interventions and targets in the lifestyle intervention group

Risk factor definition	Target and intervention
Overweight: BMI ≥ 27 kg/m^2^	10% body weight reduction is targeted. Treatment consists of additional consultations with a dietician and a physical fitness and activity programme
Physical inactivity: less than 3,5 h of merely vigorous physical activity per week and limitations during activity	> 3.5 h of physical activity per week is targeted. This is supported by a physical fitness and activity programme guided by a physiotherapist or sport cardiologist
Excessive alcohol consumption: > 8 units a week for women and > 14 units a week for men	We aim for a reduction to ≤ 8 units a week for women and ≤ 14 units a week for men. This is supported by behaviour counselling
Tobacco consumption	Patients should quit smoking. A smoking cessation programme is conducted over 6 weeks by a certified nurse
Hypertension: > 130/90 mmHg	Target blood pressure is < 130/90 mmHg. The lifestyle interventions above and antihypertensive medication are used
Hypercholesterolemia: LDL-cholesterol > 2.6 mmol/L or total cholesterol > 5.0 mmol/L	Target LDL-cholesterol is ≤ 2.6 mmol/L and total cholesterol ≤ 5.0 mmol/L. Above lifestyle interventions and lipid lowering medication are used
Glycaemic control: DM de novo is suspected in patients with HbA1c > 48 mmol/mol and fasting plasma glucose > 7.0 mmol/L	In known DM target HbA1c was < 53 mmol/mol. If DM de novo is suspected, patients are referred to their general practitioner for further treatment. Above lifestyle changes are used. Management is done by treating physician
OSA screening with home sleep apnoea test (WatchPAT™ 300). OSA was suspected if AHI ≥ 5 per hour	Referral to pulmonologist for further OSA diagnosis and treatment

*AHI* apnoea–hypopnoea index, *BMI* body mass index, *DM* Diabetes mellitus, *LDL* low-density lipoprotein, *HbA1c* glycohemoglobin, *OSA* obstructive sleep apnoea

### Lifestyle counselling and education in the intervention group

The AF nurses will collaborate with multiple specialists, such as the electrophysiologist, the sport cardiologist, the dietician and the general practitioner. To achieve successful behavioural changes, consultations will follow the principles of shared decision-making and measurable targets will be set. Patients’ knowledge levels will be reinforced through in-person educational sessions and additional digital content, disseminated via an e-health platform (Philips Engage), tailored and calibrated for this new outpatient clinic. Through the platform, patients may also report AF symptoms, submit home measurements to hospital staff, and easily communicate with staff.

### Usual care

In the usual care group, patients will visit the cardiologist and receive standard treatment according to current guidelines. The cardiologist verifies the patient’s eligibility for ablation and may start additional interventions, such as hypertension management and refer high-risk patients for OSA diagnosis and treatment.

### Ablation

Patients from both study groups will undergo the same ablation procedure with a PFA (Pulsed Field Ablation) catheter (Farawave, Farapulse Inc.). Prior to ablation, patients will use uninterrupted anticoagulation during 4 weeks and are on nil per os 12 h prior to the procedure. The ablation will be performed under conscious sedation with fentanyl, benzodiazepines and propofol. After acquiring ultrasound-guided access to the right femoral vein, the operator will perform a transseptal puncture with a steerable sheath. Patients will be heparinized with an activated clotting time target above 300 s. Fluoroscopy will be used to optimize the PFA catheter position in the pulmonary veins. After the electroporation application, entrance block will be confirmed using the Farawave catheter. In patients with recurrent typical atrial flutter besides AF, an additional cavotricuspid isthmus ablation is performed with a radiofrequency catheter.

### Post-ablation care

Patients of either treatment group will visit the outpatient clinic at 3 months after ablation. At this point, the treating physician will stop any current antiarrhythmic medication. If the patient does not experience any symptoms after ablation, no additional rhythm assessment will be performed, since this will not have therapeutical consequences. In symptomatic patients, arrhythmias will be objectivated with electrocardiogram or multiple holter assessments. If an arrhythmia is confirmed on electrocardiogram or holter after 3 months after the index ablation, patients are offered to recontinue antiarrhythmic medication and to undergo a repeat ablation. Furthermore, patients suffering from persistent AF will undergo cardioversion.

### Endpoints

The primary composite endpoint of the study is the number of hospitalizations for re-ablation and cardioversion from baseline to 12 months after ablation. The conditions for performing a re-ablation and cardioversion are explained above.

Secondary endpoints are the number of hospitalizations for re-ablation, the number of cardioversions, the number of cancelled ablations because of overall symptom reduction, mortality, number of strokes, acute ischemic events, or hospitalizations for heart failure at 12 months after ablation.

Further secondary endpoints include change in weight, blood pressure and laboratory tests at baseline, after the lifestyle intervention (for the intervention group) and at 1 year after ablation. Furthermore, ablation success is determined at 12 months after ablation. The ablation is considered to be successful when patients can stop their antiarrhythmic drugs after 3 months, without having recurrence of symptomatic arrhythmias urging for re-ablation. The elaborate lifestyle changes might negatively affect treatment burden. Treatment burden is the perception of cumulative work a patient has to perform to manage their health, and should, therefore, be limited. Therefore, further secondary endpoints include the change in quality of life and treatment burden from baseline to before ablation, at 3 months, and 12 months after ablation. The patient’s general quality of life will be assessed with the Dutch version of the EuroQol-5D questionnaire. This questionnaire includes 5 health-state measures and is suitable to determine quality-adjusted life-years for a cost–utility analysis [31]. AF-specific quality of life is assessed with the Dutch version of the AF effect on Quality of Life Survey (AFEQT). This questionnaire is suitable for describing AF-related symptoms [32]. Treatment burden will be evaluated with a Dutch translation of the Treatment Burden Questionnaire.

### Data collection

Assessment of clinical endpoints will take place at hospital visits during the intake, pre-ablation and the follow-up consultations at 3 and 12 months after ablation. Quality of life data will be collected using digital questionnaires at intake, pre-ablation, 3 months after ablation and 12 months after ablation.

An overview of the study procedures is displayed in Table 3.

**Table 3:** Study procedures

Study procedure	Intake	Pre-ablation (only in interventional group)	3 months after ablation	12 months after ablation
Assessment of clinical history and clinical endpoints	x	x	x	x
Physical examination including blood pressure and weight	x	x		x
12-lead ECG	x		x	
Laboratory test	x	x		x
Quality of life using EuroQol-5D, AFEQT, Treatment Burden Questionnaire	x	x	x	x

*ECG* electrocardiogram

### Statistical analyses

Descriptive statistics will be used to report baseline characteristics of the study population. The primary and secondary endpoints can occur during the entire participation period, which is from baseline to ablation (in the intervention group during the risk treatment; in the control group when patients await the ablation) and in 12 months after the ablation. The different pre-ablation durations amongst patients lead to different study participation periods. Therefore, the number of events will be normalized using the participation period and will be reported in numbers per year (the event rate). The clinical hypothesis that the intervention group will have a lower event rate will be tested by fitting a Poisson regression model to the event data and testing the effect of the intervention group using a Wald test. Results will be reported in terms of incidence risk ratios with accompanying 95% confidence intervals. The primary analysis will be on an intention-to-treat basis. Subgroup analyses will be performed on the primary endpoint. Covariates of interest in this study include gender, age, type of AF (paroxysmal vs. persistent) and number and extent of the respective modifiable risk factors as defined in Table 2.

The secondary endpoints will be analysed by means of chi-squared test or Fisher’s exact test depending on the number of occurrences. Changes in weight and blood pressure will be compared using the appropriate test (unpaired *t* test or Mann–Whitney *U* test), depending on the distribution of data. Laboratory tests will be analysed for differences by fitting linear mixed models to the data.

A cost-effectiveness analysis will be included, covering incremental cost-effectiveness ratio (ICER) and an evaluation of change in patient value, using the EuroQol-5D and AFEQT questionnaires and healthcare-related costs of intervention. Differences in medical hospital costs for both study groups will be compared using the appropriate statistical test (unpaired *t* test or Mann–Whitney *U* test), depending on the distribution of data. Cost savings associated with the study intervention will be calculated by subtracting the cost of the intensive treatment from the cost of the conventional treatment. The completed EuroQol-5D, AFEQT and TBQ questionnaires will be analysed for differences between treatment groups at different moments by fitting linear mixed models to the data.

A *p* value < 0.05 will be considered statistically significant and all analyses will be performed using SPSS 29 (IBM SPSS Statistics 29.0, Chicago, IL, USA). We will use complete case analysis when the percentage of missing data is less than 5%. Otherwise, we will use multiple imputations by full conditional specification.

### Sample size

The number of hospital admissions in the control group is estimated as 1.0 per patient per year [33, 34]. The proposed intervention is expected to reduce the primary endpoint with 30%. Taking the pre-ablation period into account, in the intervention group, we expect an overall event rate of 0.75 per patient per year. Assuming a balanced design with equal allocation of patients to the two groups, the minimum sample size to achieve an 80% power with 5% Type I error on the primary analysis is 144 (72 per treatment group). A total of 150 patients will be enrolled in this study, taking dropouts into account.

## Summary

This POP trial will be the first study to test the effectiveness of a nurse-led clinic applying personalized risk factor management in a prospective randomized controlled trial on ablation outcomes. The trial is designed as a comparative effectiveness trial with the purpose of directly improving AF patient care by comparing a new treatment strategy with routine clinical care [35]. Current evidence is still limited to observational data or single risk factor treatment trials. The intervention includes screening for OSA and other AF-lifestyle factors, personalized lifestyle optimization, and the use of an e-health platform. The POP researchers hypothesize that in a high-risk population with at least one lifestyle AF-risk factor, the implementation of an integral AF management clinic is beneficial for ablation outcomes. Furthermore, the POP-trial will provide more conclusive evidence on the implementation of a multidisciplinary team in AF management.

## Conclusion

The POP study will evaluate the efficacy of a multidimensional lifestyle intervention treatment before ablation in patients with paroxysmal or persistent atrial fibrillation. The study will analyse the effect of this elaborate treatment on the number of hospitalizations for re-ablation and cardioversion.
